# Highlighting the classical MRI findings in transient global amnesia

**DOI:** 10.1259/bjrcr.20190111

**Published:** 2020-09-29

**Authors:** Stephanie Vella, Reuben Grech

**Affiliations:** 1 Basic Specialist Trainee, Medical Imaging Department, Mater Dei Hospital, Msida, Malta; 2 Consultant Radiologist, Medical Imaging Department, Mater Dei Hospital, Msida, Malta

## Abstract

Transient global amnesia (TGA) is a disorder characterised by a temporary, reversible disruption of short-term memory. While the diagnosis of TGA is based on its clinical features, neuroimaging is important to exclude other sinister causes of global amnesia. Furthermore, classical MRI changes in TGA have been well described in the literature. These consist of unilateral or bilateral punctuate areas of hyperintensity in the hippocampal cornu ammonis 1 (CA1) region on diffusion-weighted imaging.

We describe a case of a 61-year-old gentleman, presenting with symptoms of transient memory loss and confusion. A stroke was initially suspected in view of his multiple risk factors. Timely MRI demonstrated the typical findings associated with TGA. Recognition of these imaging features is of the utmost importance for radiologists in order to allow for an accurate diagnosis and differentiation from ischaemic pathology.

## Case presentation

A 61-year-old gentleman presented to the Mater Dei Hospital emergency department following a short episode of transient memory loss. According to his wife, he went to work as usual that morning, however on returning home in the afternoon he was forgetting a number of recent events and was asking a number of questions repeatedly. There were no features of retrograde amnesia, with the patient still being able to recall his identity, recognise his family members and carry out usual functions. This lasted for about 30 min, after which his memory slowly returned; however, he could not remember what had happened during this episode. He denied any associated headache, weakness, speech problems or sensory disturbance. There was no witnessed loss of consciousness or seizure activity. The patient also denied any recent stressful event or head injury and this was the first such episode of memory impairment.

His medical history was notable for hypertension, diabetes and a 20-pack-year history of smoking. There was no previous history of transient ischaemic attacks, strokes or migraine. On examination, he was fully alert, oriented and cognitively intact. Parameters including blood pressure were normal and full neurological examination was unremarkable.

## Investigations

**Figure 1.**
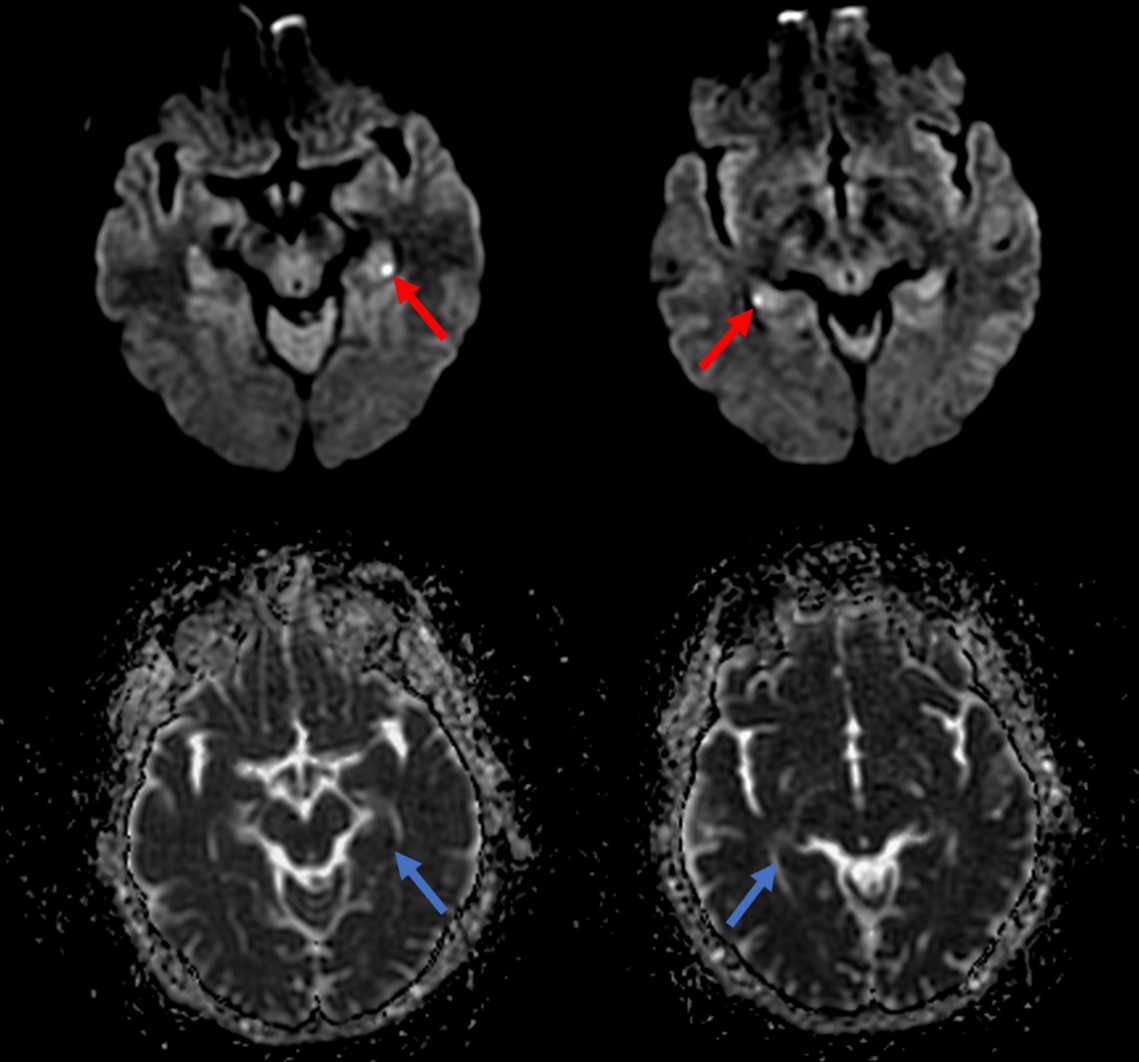
Selected axial diffusion-weighted imaging (*b* = 1000, 4 mm slice thickness) MR images (above) and corresponding apparent diffusion coefficient (ADC) maps (below) demonstrating bilateral punctate hyperintensities in the CA1 region of the hippocampi (red arrows) with corresponding signal loss on ADC (blue arrows).

**Figure 2.**
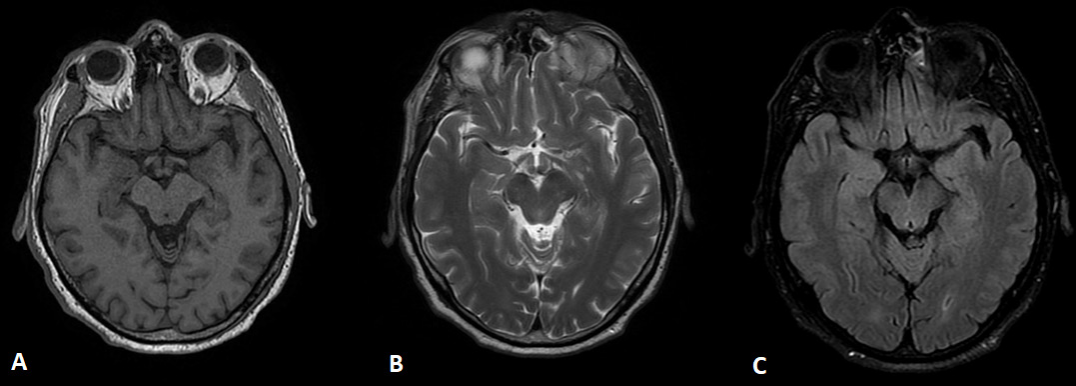
Corresponding axial T1 (panel A), T2 (panel B) and FLAIR (panel C) MR images at the level of the hippocampi. No abnormalities demonstrated.

Blood investigations were within normal limits and a CT brain showed no abnormalities. An electrocardiogram showed normal sinus rhythm. In view of his risk factors, the working diagnosis was that of an ischaemic stroke and he was admitted for further investigation and started on antiplatelet therapy. Echocardiogram and carotid Doppler showed no abnormalities. MRI of the brain using standard stroke protocol (consisting of axial T2 and fluid-attenuated inversion recovery (FLAIR), axial diffusion-weighted imaging (DWI) and corresponding apparent diffusion coefficient (ADC) map, time-of-flight MR angiography, susceptibility-weighted imaging and axial isotropic T1-weighted sequences) was performed approximately 26 hours from onset of symptoms. This revealed normal gray-white matter differentiation, with no mass lesions and normal flow voids. Note was made of punctate hyperintensities in the CA1 region of the hippocampi bilaterally on DWI [Figures 1 and 2].

### Outcome and follow-up

The case was discussed at the neurology multidisciplinary team meeting, where it was agreed that the clinical and MRI findings supported the diagnosis of transient global amnesia (TGA). The patient remained well and was discharged with no driving restrictions or need for imaging follow-up.

## Discussion

Transient global amnesia is a clinical disorder characterised by sudden onset anterograde amnesia with temporal disorientation and iterative questioning due to inability to form new memories. Long-term memory, language, visual-spatial orientation and executive functions are typically preserved, with no other associated focal neurological deficit. Episodes characteristically resolve completely within 24 hours, with return to baseline cognition. Retrograde amnesia is variably present.^1^

This syndrome usually occurs between the fifth and seventh decade of life and there is no significant gender predilection. The reported incidence is of 5–10 per 100 000 population. Cited precipitating factors include emotional stress, physical exertion, pain and the Valsalva manoeuvre.^2^

While this is a well-described disorder, the exact aetiology of this condition remains obscure. Various theories have been postulated, including venous congestion (secondary to retrograde cerebral venous blood flow),^3^ focal ischaemia, migraine as well as epilepsy. None of these however clearly explain all the condition's characteristics.^4^

The diagnosis of TGA is based on its clinical features. Criteria, such as those established by Hodges and Warlow, serve as guidance.^5^ MRI is nonetheless recommended to exclude stroke or structural pathology, where there is diagnostic uncertainty. While imaging in such cases is usually normal, it is now well-known that TGA may be accompanied by characteristic MRI changes involving the hippocampus. Structurally, the cornu ammonis or hippocampus proper is divided into four distinct zones, based upon their sensitivity to hypoxia and histological differences. CA1 (Sommer’s sector) is known as the vulnerable zone, CA2 and CA3 as the resistant sector and CA4 as the medium vulnerability sector.^6^ Classic DWI findings in TGA consist of unilateral or bilateral small punctuate hyper-intense lesions in the CA1 region of the hippocampal cornu ammonis. These changes were first described in 1998 by Strupp et al.^7^ Since that time, various studies have been carried out and shown that certain factors may improve the visibility of these findings on MRI. These include high-resolution DWI, higher B-values, thin slice thickness (2–3 mm) and a delay of 48–72 h between symptom onset and scanning.^8,9^ In fact in cases where the initial MRI performed within 24 h is normal, however clinical suspicion for TGA remains high, repeat imaging is recommended.^10^

The localisation of this pathological process to the CA1 area on imaging may give an indication to its causation. It is understood that neurons in this region of the hippocampus are particularly susceptible to metabolic stress, suggesting that TGA may result from a physiological temporary inhibition of memory formation.^11^ Interestingly, the fact that DWI changes in TGA do not appear in the hyperacute phase, unlike the restricted diffusion seen in ischaemia, further supports the theory that these imaging findings are due to an entirely different pathological process. These lesions have been noted to resolve on follow-up MRI performed after 6–12 months.^12^

Differential diagnoses for abnormal signal intensity in the hippocampi include posterior circulation ischaemia, limbic encephalitis and protracted seizures. These conditions, besides being readily distinguishable clinically, can also be distinguished radiologically to a certain degree. While in TGA, the signal hyperintensities seen on DWI are punctate and located laterally within the hippocampi, in posterior circulation infarcts the hippocampal lesions are larger in size with associated additional areas of restricted diffusion seen in the posterior cerebral artery territory, such as the occipital lobe, thalamus and corpus callosum. In protracted seizures, besides hippocampal signal abnormalities, additional changes may occur in the pulvinar and cortex. Furthermore, limbic encephalitis involves other limbic structures besides the hippocampus, such as the parahippocampal gyrus, cingulate gyrus and insula.^13^

No treatment or driving restriction is required and the condition does not usually recur in the majority of cases. Outpatient follow-up with additional imaging if clinically indicated is the mainstay of management. Several studies have shown that there is no difference in prognosis or cognitive function in patients with and without DWI changes. This also applies to comparison of patients with TGA with age-matched controls.^14^ Of note some studies have raised the possibility of an association between recurrent TGA and primary progressive aphasia, a rare neurological disorder characterised by progressive language impairment.^15^

In conclusion, the recognition of imaging findings in transient global amnesia is essential for radiologists in order to avoid such patients being misdiagnosed with ischaemia or other pathology, especially in cases where the clinical picture is atypical.

## Learning points

- While the diagnosis of transient global amnesia is based on its clinical features, neuroimaging is important to exclude other sinister causes of global amnesia.
- Classic diffusion-weighted imaging findings in TGA consist of unilateral or bilateral small punctate hyper-intense lesions in the CA1 region of the hippocampal cornu ammonis.
- Recognition of this sign is of the utmost importance for radiologists in order to allow for an accurate diagnosis and differentiation from ischaemic pathology.
